# Infant Mortality

**Published:** 1873-01-01

**Authors:** 


### INFANT MORTALITY.

During the past few months, the subject of mortality among young children, especially in our large cities, has been brought prominently to the notice of the profession, in connection with suggestions for lessening it. Dr. J. M. Toner, of Washington, D. C., by referring to the census returns, has shown that nearly fifty per cent. of the gross mortality in the larger cities of our country occurs in children under five years of age. And every observing practitioner knows that much the larger part of this mortality takes place during the hot months of summer, and in children under three years. Dr. Toner's statistics also show that the ratio of infant mortality in the country districts is so much less, especially during the summer months, that he suggests, as an important sanitary measure, the removal of young children, with their mothers and nurses, from the cities into elevated and healthy country districts, during the two months of highest summer heat. To make this practicable for the poor and laboring classes, whose children suffer most, he suggests the establishment of public sanitary parks or encampments, furnished with tents, barracks, and other necessary conveniences, and located as near our large cities as healthy and accessible places can be found. It is proposed that these parks or encampments shall be provided with all the needed accommodations by the municipal authorities of cities, aided by benevolent individuals, that they may be *free* to all who have feeble or sick children, however poor they may be. It is thought that 1,500 or 2,000 acres of elevated ground—covered, in part at least, with trees, under-drained, and well supplied with pure water—would be sufficient as a summer resort for the children of the working classes of any one of our large cities. An abundant supply of fresh, pure milk would also be a necessity; and the whole should be under the supervision of a good physician and three or four efficient sanitary policemen.

Theoretically, the scheme thus outlined looks fair and practicable. The subject is one of the most important that can engage the attention of the social economist and the physician. The terrible loss of life in infancy in all our great commercial cities, during the summer months, is painful to contemplate. And it is quite time that the medical profession entered sincerely upon the work of devising means for permanently lessening the evil. Whether the measure proposed by Dr. Toner is the best that can be devised, or whether it should constitute one of a series of measures, all aiming to facilitate the accomplishment of the same object, we are not prepared to state at present, but will return to a further consideration of the subject at a future time.

